# Combined preoperative albumin-bilirubin (ALBI) and serum γ-glutamyl transpeptidase (GGT) predicts the outcome of hepatocellular carcinoma patients following hepatic resection

**DOI:** 10.7150/jca.33877

**Published:** 2019-08-27

**Authors:** Chi-Hao Zhang, Xiao-Chun Ni, Bi-Yin Chen, Shuang-Jian Qiu, Yi-Ming Zhu, Meng Luo

**Affiliations:** 1 Department of General Surgery, Shanghai Ninth People's Hospital, School of Medicine, Shanghai Jiao Tong University, Shanghai, China; 2 Department of Oncology, Shanghai Ninth People's Hospital, Shanghai Jiao Tong University School of Medicine, Shanghai, China.; 3 Department of General Surgery, Zhongshan Hospital, School of Medicine, Fudan University, Shanghai, China

**Keywords:** Hepatocellular carcinoma, ALBI, GGT, Cox analysis, nomogram

## Abstract

**Background**: Liver function is an important prognostic factor for patients with hepatocellular carcinoma. The purpose of this study was to develop and validate a nomogram integrating the albumin-bilirubin (ALBI) and serum γ-glutamyl transpeptidase (GGT) level to predict postoperative overall survival (OS) and disease-free survival (DFS) of hepatocellular carcinoma (HCC).

**Methods**: The effect of combined of ALBI and GGT on HCC prognosis was investigated using univariate and multivariate Cox analyses. The nomogram for OS and DFS were developed, respectively, and their predictive ability was compared with other conventional staging systems, including the American Joint Commission on Cancer (AJCC), Barcelona Clinic Liver Cancer (BCLC) and the Cancer of the Liver Italian Program (CLIP).

**Results**: Combined ALBI and GGT was highly associated with OS (*P*<0.001) and DFS (*P*<0.001) of HCC patients treated with hepatic resection. In addition, the C-index of the OS (0.706±0.034) or DFS (0.674±0.032) nomogram in the training cohort was larger than AJCC, BCLC and CLIP. The Akaike information criterion (AICs) of the OS (2178.405) or DFS (2961.018) nomogram in the training cohort was smaller than above staging systems. The results suggested that the OS or DFS nomogram was the most powerful model to predict HCC prognosis. The similar trend was observed in the validation cohort.

**Conclusion**: The novel nomogram integrating ALBI and GGT was highly associated with OS and DFS of postoperative HCC patients.

## Introduction

Hepatocellular carcinoma (HCC) is one of the most common and aggressive malignant tumors and is ranked third among causes of cancer-associated death in the world 1. To date, surgical resection is still a first alternative for primary HCC patients, especially for those with enough residual liver function. But the survival time of these patients is not ideal even after the resection because of the high relapse rate. A number of studies have shown that there are approximately 50% to 70% HCC patients, even in early stage, would have tumor recurrence 5 years after surgical removal 2, 3. Therefore, the factors that have an effect on the long-outcome of HCC patients receiving operation have been studied in order to improve the prognosis.

It has been reported that liver function is highly associated with the clinical outcome and therapeutic choices. Poor liver function always indicates a high risk of surgery and unsatisfactory prognosis 4, 5. Currently, Child-Pugh (C-P) system has been widely applied in clinical practice for assessment of liver function. However, there are some limitations which may result in inaccurate evaluation of liver function. For example, it is need to assess two subjective variables including ascites and encephalopathy when we use C-P system, which makes it difficult to determine the degree of the symptoms. In addition, the same weighting is allocated on the five clinical factors 6, 7. To overcome these limitations, the albumin-bilirubin (ALBI) grade was developed in 2015 to evaluate hepatic function of HCC patients 6. Several studies have demonstrated that ALBI grade is a powerful tool in the prediction of survival time of HCC patients after surgery. Furthermore, the Barcelona Clinic Liver Cancer (BCLC) system or the Cancer of the Liver Italian Program (CLIP) score has similar or improved accuracy in predicting HCC patients' survival when ALBI grade is taken into account 8, 9.

Expect for hepatic function, some investigations have confirmed the important role played by tumor biomarkers in the surveillance of HCC patients' survival outcome. Zhou suggested in their investigation that the measurement of GGT level had value in the diagnosis of HCC. In addition, Yang and his colleagues found the presence of HCC should be considered when GGT increased, AST/ALT>1 and GGT>ALT>1^10^. In accordance with the above results, our previous study also revealed that GGT level was a potential prognostic factor in HCC patients after hepatic resection. Recently, another study by Dong et al found that the combination of the ALBI grade and GGT level could significantly predict the overall survival and recurrence-free survival of postoperative HCC patients 11. However, the role of this combination variable in all possible prognostic factors has not been illustrated.

In this study, we aimed to seek the associations between this combination variable and overall survival (OS) as well as the recurrence of HCC patients receiving hepatic resection. The OS and DFS nomogram were then built on a basis of the ALBI grade and GGT level for patients following surgery. These two nomograms would provide more accurate information about prognostic prediction and help clinicians better understand the influence of ALBI and GGT on HCC survival and relapse.

## Methods

**Patients.** Patients who received radical surgery for primary HCC were selected from Zhongshan Hospital, Fudan University (Shanghai, China). Patients in the cohort from were followed up between 2011 and 2017 until outcome event occurred or study ended. The inclusion criteria for selected patients were as follows: (1) patients with primary HCC diagnosed microscopically, for whom curative resection was performed; (2) aged over 18; (3) complete patient follow-up information; (4) laboratory test data was available and reliable; (5) no evidence of distance metastases or hilar lymph node metastases; (6) no history of having preoperative anti-cancer treatment. Patients were excluded if important data was not available for constructing different staging systems. The patients were randomly divided into training cohort (2/3) and validation cohort (1/3) through setting random seed in R software (version R-3.4.3, the R Foundation for statistical computing). This study was approved by all participating hospitals. Each patient was followed up every 3-6 months and had routine examination, such as serum alpha-fetoprotein (AFP), abdominal CT and blood biochemistry.

### Data collection

Patients' basic characteristics (gender, age), pretreatment laboratory indexes (liver function, alpha-fetoprotein (AFP), hepatitis B serology and etc.) and part of tumor-related information (tumor size, tumor number, microvascular invasion) were acquired at time of diagnosis. The rest tumor characteristics, such as differentiation and tumor capsule, were recorded according to the findings during surgery.

The ALBI score was calculated according to the following formula: 0.66 × log 10 (total bilirubin μmol/L)-0.085 (albumin g/L). We stratified the patients into three groups on a basis of specific cut-off values described in previous literature: ALBI grade 1(≤-2.60), group 2 (-2.60 ~ -1.39) and group 3 (≥-1.39). The GGT was divided into high and low group with 50 U/L as a cut-off point. The novel parameter was then created and classified into three scores based on preoperative ALBI (≤-2.60 and >-2.60) and GGT (≤50 and >50) 11: Ⅰ: both of them were low, Ⅱ: either ALBI or GGT was high or low, Ⅲ: both of them were high.

In this study, OS and DFS were used as the primary endpoint. And OS was defined as the time interval from the date of surgery to the date of death of any cause or last follow-up. While DFS was defined as the time interval from the date of surgery to the date of recurrence of HCC or last follow-up.

### Statistical methods

Continuous variables were displayed as median with range and the differences between groups were compared using the Mann-Whitney test. The chi-square test or Fisher's exact test was applied to categorical variables to identify associations between groups. The Kaplan-Meier method was used to determine the survival curves and the log-rank test was used to test the survival differences. Univariate analysis was performed in the training cohort to identify variables related with survival outcomes. Any variables with *P* value less than 0.05 in the univariate analysis were further analyzed using multivariate Cox regression model. The variables, which remained to be significant in the multivariate analysis, were taken for constructing nomogram by using rms package in R software. Then calibration was plotted in the validation cohort to assess the robust of the nomogram. In addition, the concordance index (C-index), as well as corrected Akaike information criterion (AICs), was calculated to estimate and compare the prognostic predictive ability among different staging systems. A larger value of C-index shows that the prediction model is more accurate, whereas a smaller value of AICs is an indicator of a better model for prognostic prediction. DCA was used for assessment of the clinical significance of the nomogram. The differences were regarded as significant when the P value was less than 0.05 (two-sided). All the analyses were performed using R software.

## Results

### Patient characteristics

According to the above inclusion and exclusion criteria, a total of 714 patients were included in our study. Patients were randomly divided into two cohorts using computational method. One consisting of 520 subjects was used as a training cohort and the other as a validation cohort.

The baseline characteristics of the training cohort and the validation cohort are summarized in Table 1. The training cohort consisted of 520 patients with a median age (range) of 54 years old (21-92). The number of females was significantly less than the number of males (16% vs. 84%). Most of the patients had hepatitis B induced liver cirrhosis. Usually, the patients in the training cohort had a single tumor (85.8% vs. 14.2%) with diameter less than 5cm (64.8% vs. 35.2%), no microvascular invasion (69.4% vs. 30.6%), poor differentiation (23.2% vs. 76.8%) and no complete tumor capsule (62.7% vs. 37.3%). Regarding laboratory parameters, 382 (73.5%) patients had AFP higher than 400ng/ml and 266 (51.2%) had GGT serum level less than 50 U/L. The median serum level of ALT was 42.6 U/L (range, 3.0-615.0 U/L). As all patients have underwent surgical procedure, therefore, there were no patients classified as Child-Pugh C or ALBI grade Ⅲ. The detailed information about the patients in the validation cohort was list in Table 1. In summary, no significant differences in variables were found between the training cohort and the validation cohort.

### Relationship between ALBI-GGT score and clinicopathological parameters

Correlations between the ALBI-GGT score and clinicopathological parameters were shown in Table 2. We found that the ALBI-GGT score was significantly associated with degree of cirrhosis (*P*<0.001), tumor size (*P*<0.001), tumor number (*P*=0.020), vascular invasion (*P*<0.001), differentiation (*P*<0.001) and other staging systems (*P*<0.001). However, this score was not related with age, gender, ALT serum level, AFP concentration, tumor capsule and Child-Pugh grade.

### Survival analysis

**Figure 1:**
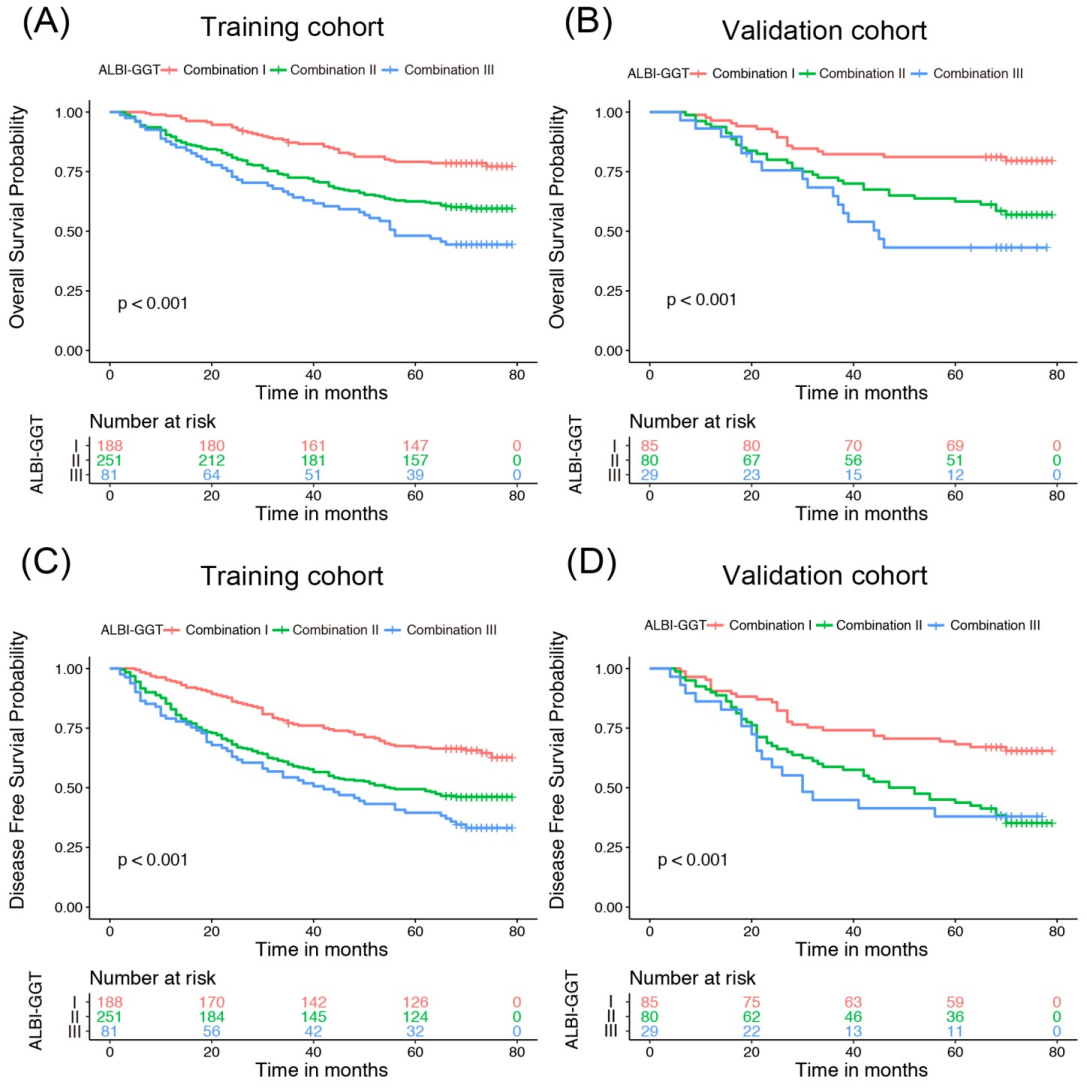
Kaplan-Meier curve of HCC patients according to ALBI-GGT scores. Kaplan-Meier curve of overall survival for HCC patients according to ALBI-GGT scores in training cohort (A) and validation cohort (B). Kaplan-Meier curve of disease-free survival for HCC patients according to ALBI-GGT scores in training cohort (C) and validation cohort (D).

To explore whether ALBI-GGT score has an influence on prognosis of HCC patients receiving surgical resection, the OS and DFS curves were plotted according to the ALBI-GGT score (Figure 1). We found that survival time and recurrence time shortened in the training cohort with an increasing ALBI-GGT score. Patients with higher ALBI-GGT score had worse survival prognosis compared with patients with lower ALBI-GGT score. The similar trend was also observed in the validation cohort. The 5-year OS and DFS rates in the training cohort were 78.19%, 62.55% and 48.15% vs. 67.02%, 49.40% and 39.51% for patients with ALBI-GGT combination I, II and III, respectively (*P*<0.001). While in the validation cohort, the 5-year OS rates were 81.18%, 63.75% and 41.37% vs. 69.41%, 45.00% and 39.93% for patients with ALBI-GGT combination I, II and III, respectively (*P*<0.001). Univariate analysis was conducted and showed that the degree of cirrhosis (*P*=0.005), AFP concentration (*P*=0.038), tumor size (*P*<0.001), tumor number (*P*=0.002), vascular invasion (*P*<0.001), differentiation (*P*=0.001), Child-Pugh grade (*P*=0.044) and ALBI-GGT score (*P*<0.001) were potential prognostic factors for overall survival. Likewise, we found that the degree of cirrhosis (*P*=0.001), tumor size (*P*<0.001), tumor number (*P*<0.001), vascular invasion (*P*<0.001), differentiation (*P*<0.001) and ALBI-GGT score (*P*<0.001) were significant indicators for DFS (Table 3).

**Figure 2:**
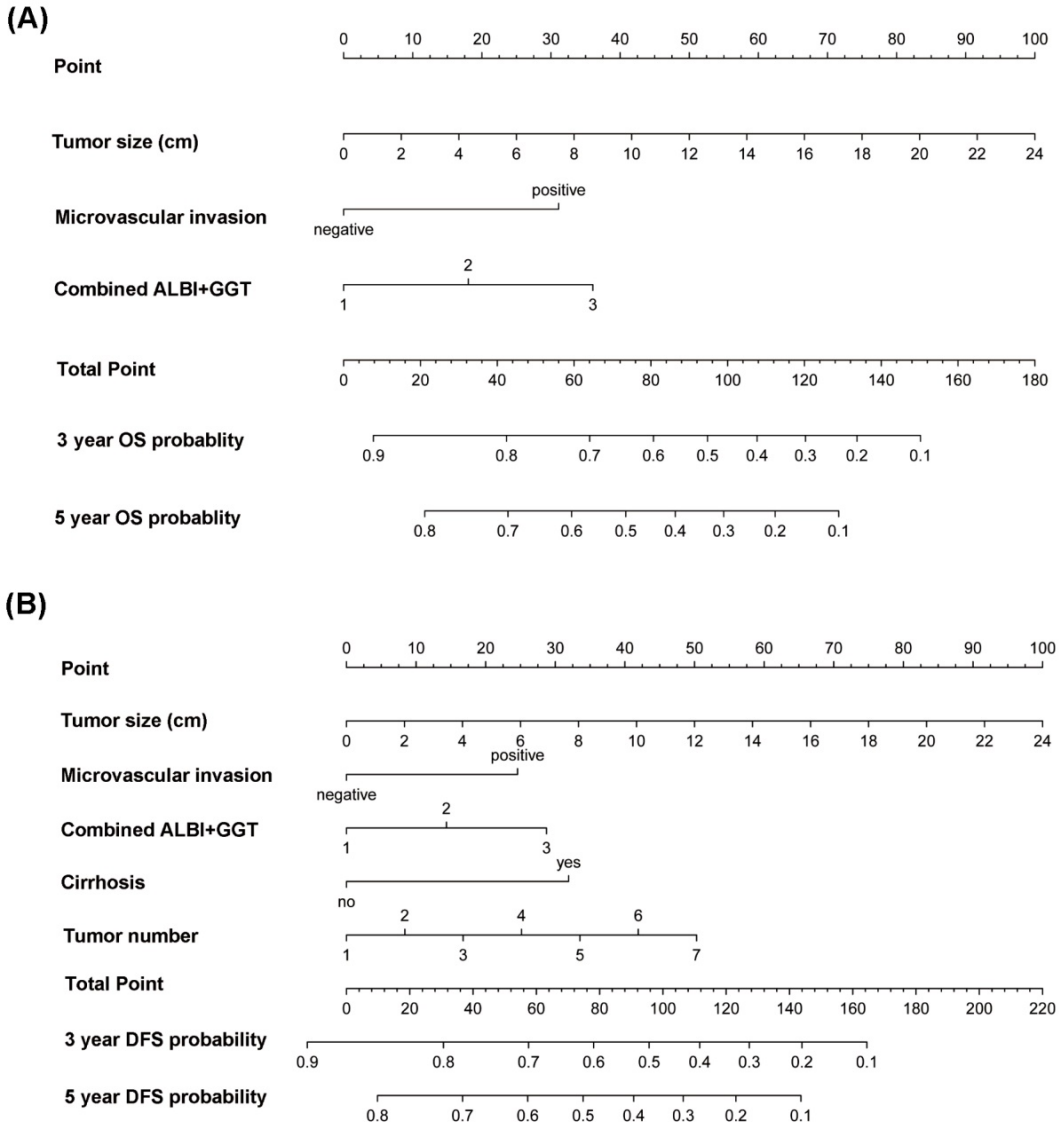
Nomogram for predicting overall survival (A) and disease-free survival (B) of hepatocellular carcinoma patients.

In addition, multivariate Cox regression analysis was performed to further identify the factors related with OS and DFS. According to the results of univariate analysis, we selected the indicators with *P* less than 0.05 and put them into the multivariate Cox model. The result showed that tumor size, vascular invasion and ALBI-GGT score (all *P*<0.05) were significantly associated with patients' survival time. We found that the degree of cirrhosis, tumor size, tumor number, vascular invasion and ALBI-GGT score (all *P*<0.05) were independent factors in the prediction of disease-free survival (Table 3). Moreover, the nomograms were built on a basis of the independent risk factors of OS and DFS, respectively (Figure 2).

### Predictive Performance of the Nomogram in the training cohort

**Figure 3:**
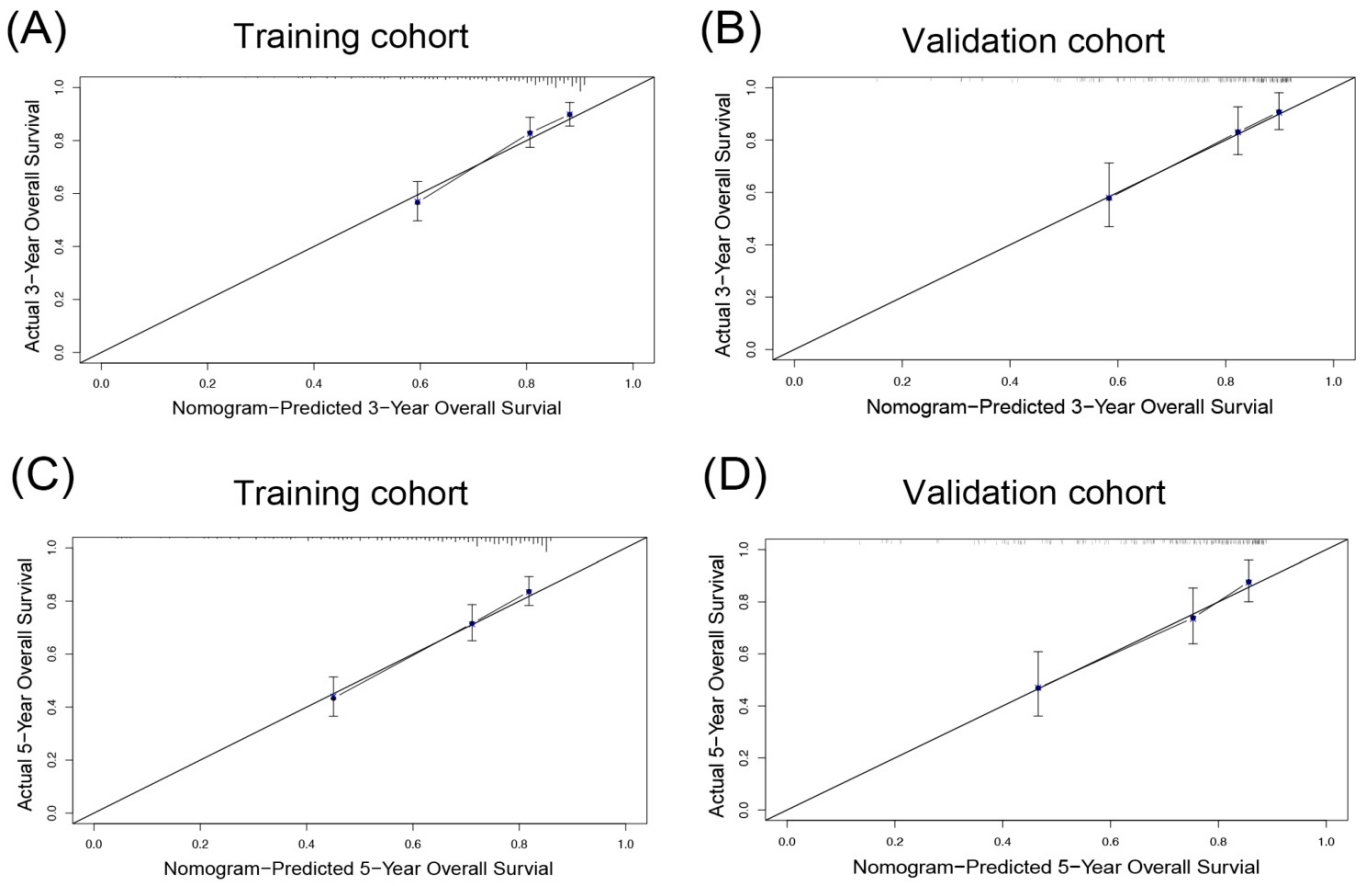
The calibration plot for OS prediction. The calibration curves for predicting 3- (A and B), 5- (C and D) overall survival (OS) rates of patients with hepatocellular carcinoma in the training cohort (A, C) and the validation cohort (B, D), respectively.

As shown in Table 4, the AICs of the OS nomogram was 2178.405, which was smaller than that of AJCC (2220.506), BCLC (2194.612) and CLIP staging system (2211.851). Furthermore, the OS nomogram showed the largest C-index (0.706, 95%CI: 0.672-0.740) in comparison with other three staging systems. On the other hand, calibration curves were plotted to test the performance of the nomogram in the prediction of OS. The result showed good agreement between the model prediction and actual observation in terms of 3- and 5-year OS (Figure 3A, C), suggesting the predictive model for OS had the greatest consistency with the actual observation.

**Figure 4:**
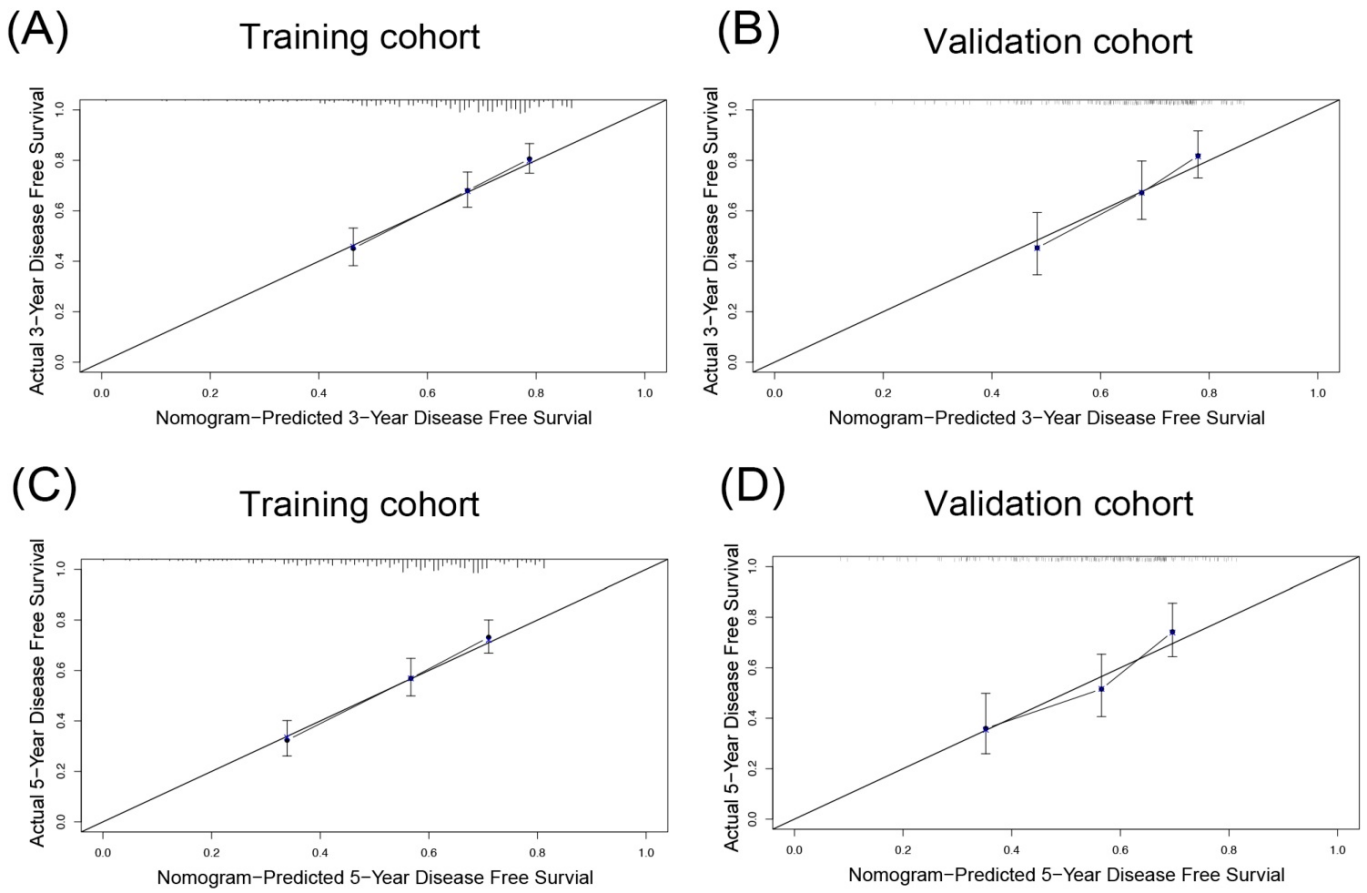
The calibration plot for DFS prediction. The calibration curves for predicting 3- (A and B), 5- (C and D) disease-free survival (DFS) rates of patients with hepatocellular carcinoma in the training cohort (A, C) and the validation cohort (B, D), respectively.

Similarly, the DFS nomogram had the largest C-index (0.674, 95%CI: 0.642-0.706), and the smallest AICs (2961.018) among all staging systems, suggesting the DFS nomogram was the most powerful predictive model offering the most accurate information about HCC recurrence. Compared to actual observations, nomogram predictions were consistent in predicting survival at 3 and 5 years in terms of the calibration internal validation curve for DFS in the validation cohort (Figure 4A, C).

**Figure 5:**
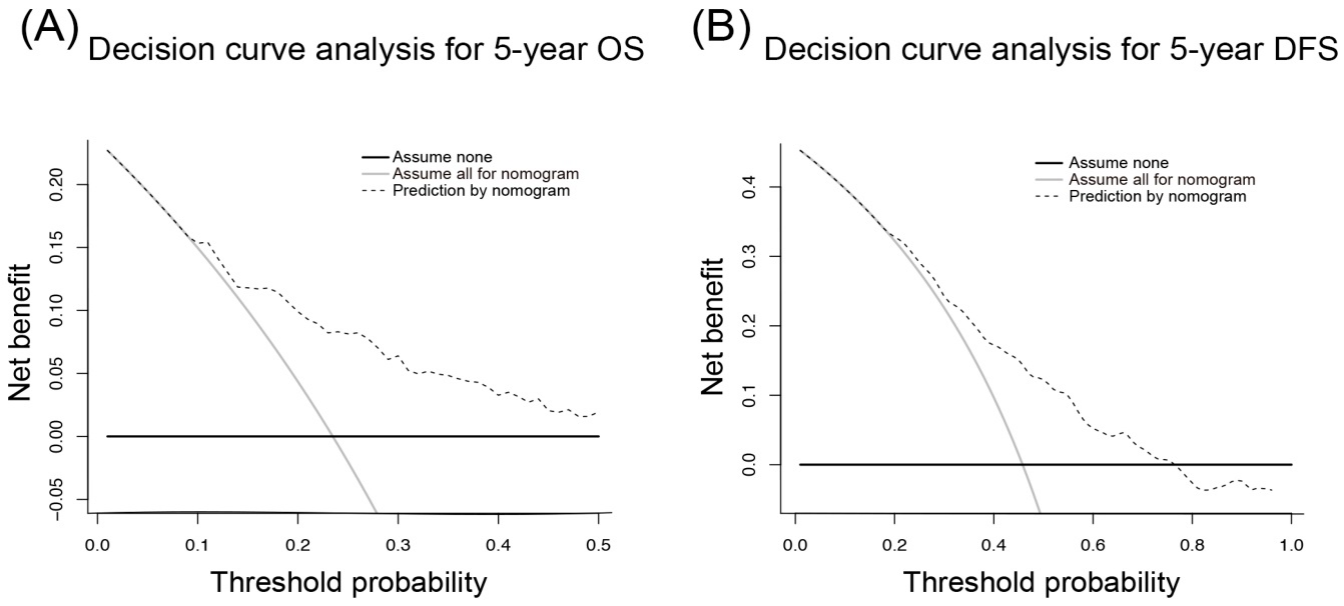
Decision curve analysis for 5-year overall survival (A) and disease-free survival (B) of hepatocellular carcinoma patients.

The DCA is a novel approach to assess the clinical utility of predictive models and showed that our nomogram had a good net benefit with a wide range of threshold probabilities and an excellent performance for predicting OS and DFS in HCC patients after resection. These results represent a good estimation of decision outcomes at high threshold probability levels (Figure 5).

### Validation of the Nomogram

In the validation cohort, the C-index of the OS or DFS nomogram was larger than other three staging systems. The AICs of the OS or DFS nomogram was demonstrated to be the smallest, indicating the nomogram in our study was a better predictive model. The calibration curves showed the prediction in the probability of 3- or 5-year OS or DFS had good agreement with observation (Figure 3B, D, 4B, D).

## Discussion

In current study, we presented an analysis of HCC patients following curative resection from a single medical center. By our analysis, the ALBI-GGT score was identified as a significant predictor of survival and tumor recurrence for patients within our criteria. In addition, we created an OS nomogram and a DFS nomogram based on our cohort, in which tumor size, microvascular invasion and the combined ALBI and GGT were integrated. While the DFS nomogram, expect for above variables, comprises another two clinical parameters, such as the degree of cirrhosis and tumor number. Both of models had superior prognostic predictive capacities for OS or DFS with high accuracy in comparison with conventional staging systems. Meanwhile, our study showed that they had an excellent performance in clinical utility.

It is particularly important to evaluate liver function in clinical practice because cirrhosis-related liver dysfunction is one of the main causes leading to death 12, 13. Currently, C-P system is widely used as a useful tool for assessment of cirrhotic patients' liver function. But there are several limitations lying in this grading system. For example, it is not easy to distinguish the degree of ascites or hepatic encephalopathy because both of them are highly subjective 14, 15. On the other hand, even the patients progress into liver dysfunction, they seldom display refractory ascites, or obvious encephalopathy 11. Thus, sometimes it seems hard to accurately assess the residual hepatic function with C-P system. Moreover, the parameters considered in this system have equal weighting and the cut-off points are arbitrary, which may cause clinicians to overestimate or underestimate the patients' actual hepatic function 8. We know that the C-P system was originally developed for cirrhotic patients to predict their prognosis. However, it is not clear whether or not the C-P system is still applicable for patients with non-cirrhotic HCC.

The ALBI grade was recently developed as a potential alternative to the C-P system 6, 16. It involves two objective parameters, such as albumin and bilirubin, which can be easily obtained by blood examination. Although its application in the clinical work was questioned in the beginning due to a lack of sufficient evidence. Nevertheless, various recent studies have demonstrated that the ALBI grade provides reliable and objective information about patients' liver function 6, 17, 18. Pinato and his colleagues 19 revealed in their investigation that ALBI score had clinically meaningful stratifying ability for each BCLC stage of HCC. Some studies also reported that ALBL grade was a prognostic predictor for postoperative HCC patients in early stage 20. Li et al found that compared with the C-P score, the ALBI grade was better at predicting the survival time of HCC patients after receiving surgery 21. More importantly, it is reported that not all patients with C-P grade A had the similar prognosis and patients in this group should not be taken as homogeneity 6. In the present investigation, patients with C-P grade A accounted for 99.6% of all postoperative HCC patients and they could be divided into two groups based on ALBI cut-off values. Patients with ALBI score 1 (n=523, 5-year OS: 70.7%) had longer survival time than those with ALBI score 2 (n=191, 5-year OS: 57.6%) (*P<0.05*). No patients were stratified into ALBI score 3. This result at least claims that it may not be appropriate to predict the outcome of HCC patients using C-P system. Despite the superiority of ALBI grade, its abilities in the prediction of tumor recurrence are limited because of a lack of other biomarkers 4, 8, 22. Among all tumor biomarkers, serum GGT level is considered as an important marker in the diagnosis of primary HCC. Some investigations have demonstrated that the elevated GGT expression could facilitate tumor progression, metastasis, and drug resistance though redox regulation, DNA damage or modulating cell apoptosis and adhesion 23-25. Moreover, it is reported that the high expression of GGT was highly associated with shortened cancer-specific survival and disease-free survival, which has been proved in several solid malignancies 26, 27. Furthermore, serum GGT level was shown to have predictive value for HCC patients following hepatic resection 28.

To the best of our knowledge, the combination of ALBI and GGT included in our nomogram is a new indicator in the prediction of survival of HCC after resection. In contrast to previous studies, which stressed the role of either the ALBI grade or the GGT in predicting HCC prognosis, our study proposed that this new indicator may improve the capabilities in prognostic prediction, especially in the tumor recurrence. Because we found the ALBI grade 1 also had high expression of GGT. By our analysis, patients under this condition had a poor outcome compared with those with ALBI grade 1 and low GGT level. On the other hand, our nomogram contained the laboratory indicators that are quite easy to be measured and calculated, such as ALBI and GGT. In addition, tumor size, microvascular invasion, cirrhosis and tumor number can be confirmed by abdominal ultrasound, computed tomography and pathological findings. Different from BCLC, CLIP staging system, all these parameters contained in our nomogram are objective and stable, avoiding the subjective judgement from the surgeons.

Given there are still many factors having influence on survival and recurrence of HCC patients, we incorporated other risk factors into the nomograms, such as tumor size, vascular invasion. Multivariate Cox analyses have shown that the combination of ALBI and GGT could be used as a survival-related or recurrence-related factor for HCC. We also calculated the C-index and AICs for our nomograms and other conventional staging systems, such as AJCC TNM, BCLC and CLIP. The lower value of AIC always indicates better fit of a statistical model, while the larger value of C-index means a more accurate model. The results revealed that our nomograms had the lowest AIC and the largest C-index, which suggested that the nomograms were the most powerful models compared with other staging systems. More importantly, DCA analyses showed that our models had the potential to be utilized in clinical work.

There are some limitations in our study. Firstly, exceeding 99% patients in our analysis were classified as Child-Pugh A. Therefore, it is not clear whether our models are equally valid in other G-P grade patients. Moreover, this study only included surgery treated patients. It is necessary to enroll HCC patients following other therapy, such as trans-catheter arterial chemoembolization. Lastly, due to unexhaustive data record, we only compared our model with TNM, BCLC and CLIP staging system, and other classifications were not included in this study for further analysis, such as MELD score. Therefore, the results of our study need to be further validated by other.

## Conclusion

Our study demonstrated that the combination of ALBI and GGT was associated with OS and DFS of HCC receiving hepatic resection. In addition, the OS nomogram or DFS nomogram provided more accurate and reliable prognostic information in comparison with traditional staging system.

## Figures and Tables

**Table 1:** Characteristics of patients with primary HCC in the training cohort and validation cohort.

Characteristics	Training cohort	Validation cohort	*p* value
Age, year, median, (range)	54.0 (21.0-92.0)	54.5 (22.0-81.0)	0.647
Gender (female/male), %	83/437 (16 /84)	27/167 (13.9 /86.1)	0.501
Cirrhosis (yes/no), %	389/131 (74.8 /25.2)	145/49 (74.7 /25.3)	0.986
ALT, U/L, median (range)	42.6 (3.0-615.0)	43.6 (9.0-915.0)	0.833
AFP, ng/ml, (≤400/>400), %	382/138 (73.5 /26.5)	138/56 (71.1 /28.9)	0.534
HBsAg (positive/negative), %	405/115 (77.9 /22.1)	153/41 (78.9 /21.1)	0.778
GGT, U/L, (≤50/>50), %	266/254 (51.2 /48.8)	88/106 (45.4 /54.6)	0.168
Tumor size, cm, (≤5.0/>5.0), %	337/183 (64.8 /35.2)	121/73 (62.4 /37.6)	0.546
Tumor number (single/multiple), %	446/74 (85.8 /14.2)	164/30 (84.5 /15.5)	0.678
Microvascular invasion (yes/no), %	159/361 (30.6 /69.4)	52/142 (26.8 /73.2)	0.326
Differentiation (poor/moderate/well), %	121/226/173 (23.2/43.5/33.3)	45/92/57 (23.2/47.4 /29.4)	0.323
Tumor capsule (yes/no), %	194/326 (37.3 /62.7)	59/135 (30.4 /69.6)	0.087
ALBI (Ⅰ/Ⅱ) , %	377/143 (72.5 /27.5)	146/48 (75.3 /24.7)	0.459
Child-Pugh grade (A/B), %	518/2 (99.6 /0.4)	193/1 (99.5 /0.5)	1.000
AJCC stage (Ⅰ/Ⅱ/Ⅲ) , %	349/112/59 (67.1 /21.5 /11.3)	130/42/22 (67.0 /21.6 /11.3)	0.986
BCLC stage (1/2/3), %	324/160/36 (62.3 /30.8 /6.9)	115/69/10 (59.3 /35.6 /5.2)	0.807
CLIP stage (0/1/2/3/4), %	292/145/45/32/6 (56.2 /27.9/8.7 /6.2 /1.2)	105/57/17/13/2 (54.1 /29.4 /8.8 /6.7 /1.0)	0.720

ALT, Alanine aminotransferase; AFP, Alkaline phosphatase; HBsAg, Hepatitis B virus surface antigen; GGT, Gamma-glutamyl transpeptidase; ALBI, Albumin-bilirubin; AJCC, American Joint Committee on Cancer, BCLC, Barcelona Clinic Liver Cancer; CLIP, Cancer of the Liver Italian Program.

**Table 2:** Comparison of characteristics of patients with different ALBI-GGT scores in entire cohort.

Variable	ALBI-GGT score 1	ABLI-GGT score 2	ABLI-GGT score 3	*P*
Age, year, median, (range)	53.7 (23.0-83.0)	54.6 (25.0-92.0)	54.1 (21.0-78.0)	0.634
Gender (female/male), %	52/221 (19.0/81.0)	45/286 (13.6/86.4)	13/97 (11.8/88.2)	0.096
Cirrhosis (yes/no), %	185/88 (67.8/32.2)	248/83 (74.9/25.1)	101/9 (91.8/8.2)	<0.001
ALT, U/L, median (range)	47.9 (10.0-915.0)	40.8 (3.0-332.0)	36.4 (7.0-252.0)	0.111
AFP, ng/ml, (≤400/>400), %	205/68 (75.1/24.9)	249/82 (75.2/24.8)	44/66 (40.0/60.0)	0.004
HBsAg (positive/negative), %	207/66 (75.8/24.2)	257/74 (77.6/22.4)	94/16 (85.5/14.5)	0.114
GGT (≤50/>50), %	273/0 (100/0)	81/250 (24.5/75.5)	0/110 (0/100)	<0.001
Tumor size, cm, (≤5.0/>5.0), %	216/57 (79.1/20.9)	186/145 (56.2/43.8)	56/54 (50.9/49.1)	<0.001
Tumor number (single/multiple), %	246/27 (90.1/9.9)	274/57 (82.8/17.2)	90/20 (81.8/18.2)	0.020
Microvascular invasion (yes/no), %	54/219 (19.8/80.2)	106/225 (32.0/68.0)	51/59 (46.4/53.6)	<0.001
Differentiation (poor/moderate/well), %	50/155/68 (18.3/56.8/24.9)	79/143/109 (23.9/43.2/32.9)	37/20/53 (33.6/18.2/48.2)	<0.001
Tumor capsule (yes/no), %	101/172 (37.0/63.0)	116/215 (35.0/65.0)	36/74 (32.7/67.3)	0.717
ALBI (Ⅰ/Ⅱ), %	273/0 (100/0)	250/81 (75.5/24.5)	0/110 (0/100)	<0.001
Child-Pugh grade (A/B), %	273/0 (100/0)	329/2 (99.9/0.1)	109/1 (99.1/0.9)	0.359
AJCC stage (Ⅰ/Ⅱ/Ⅲ), %	214/49/10 (78.4/17.9/3.7)	211/71/49 (63.7/21.5/14.8)	54/34/22 (49.1/30.9/20.0)	<0.001
BCLC stage (1/2/3), %	210/55/8 (76.9/20.1/3.0)	179/126/26 (54.1/38.1/7.8)	50/48/12 (45.5/43.6/10.9)	<0.001
CLIP stage (0/1/2/3/4), %	176/77/13/7/0 (64.5/28.2/4.8/2.5/0)	182/86/34/23/6 (55.0/26.0/10.3/6.9/1.8)	39/39/15/15/2 (35.5/35.5/13.6/13.6/1.8)	<0.001

ALT, Alanine aminotransferase; AFP, Alkaline phosphatase; HBsAg, Hepatitis B virus surface antigen; GGT, Gamma-glutamyl transpeptidase; ALBI, Albumin-bilirubin; AJCC, American Joint Committee on Cancer, BCLC, Barcelona Clinic Liver Cancer; CLIP, Cancer of the Liver Italian Program.

**Table 3:** Univariate and Multivariate analyses of prognostic factors for hepatocellular carcinoma patients in training cohort.

	Overall Survival					Disease-free Survival		
			Multivariate analysis				Multivariate analysis	
Variable	N=520	Univariate *P*	HR (95%)	*P*		Univariate *P*	HR (95%)	*P*
Age (≥65/<65)	96/424	0.464	—	NA		0.427	—	NA
Gender (female/Male)	83/437	0.101	—	NA		0.508	—	NA
Cirrhosis (yes/no)	389/131	0.005	1.428 (0.957-2.132)	0.081		0.001	1.551 (1.111-2.165)	0.010
HBsAg (positive/negative)	405/115	0.783	—	NA		0.242	—	NA
ALT, U/L, median, (range)	42.6 (3.0-615.0)	0.338	—	NA		0.988	—	NA
AFP, ng/ml, (≤400/>400)	382/138	0.038	0.972 (0.705-1.341)	0.863		0.204	—	NA
Tumor size, cm, (≤5.0/>5.0)	337/183	<0.001	1.980 (1.456-2.694)	<0.001		<0.001	1.633 (1.253-2.130)	<0.001
Tumor number (single/multiple)	446/74	0.002	1.341 (0.924-1.946)	0.123		<0.001	1.489 (1.085-2.045)	0.014
Microvascular invasion (yes/no)	159/361	<0.001	1.972 (1.451-2.679)	<0.001		<0.001	1.601 (1.227-2.091)	0.001
Differentiation	173/226/121							
well		0.012						
moderate		0.001	1.153 (0.849-1.564)	0.182		0.031	1.162 (0.895-1.510)	0.314
poor		0.004	1.382 (0.721-1.621)	0.091		0.001	1.195 (0.826-1.531)	0.121
Tumor capsule (yes/no)	194/326	0.597	—	NA		0.062	—	NA
Child-Pugh grade (A/B)	518/2	0.044	1.173 (0.866-1.589)	0.302		0.057	—	NA
ALBI-GGT	205/233/82							
ALBI-GGT (1)		<0.001				<0.001		
ALBI-GGT (2)		<0.001	1.663 (1.146-2.413)	0.007		<0.001	1.503 (1.110-2.034)	0.008
ALBI-GGT (3)		<0.001	1.947 (1.242-3.052)	0.004		<0.001	1.638 (1.119-2.398)	0.011

ALT, Alanine aminotransferase; AFP, Alkaline phosphatase; HBsAg, Hepatitis B virus surface antigen; GGT, Gamma-glutamyl transpeptidase; ALBI, Albumin-bilirubin.

**Table 4:** Comparison of prognostic performance among 4 staging systems.

		Overall Survival			Disease-free Survival	
	Staging system	Training cohort	Validation cohort		Training cohort	Validation cohort
AIC	ALBI-GGT	2178.405	617.827		2961.018	938.731
	AJCC	2220.506	628.268		2993.223	948.234
	BCLC	2194.612	622.070		2975.837	949.826
	CLIP	2211.851	626.315		2996.169	951.285
						
C-index	ALBI-GGT	0.706±0.034	0.681±0.042		0.674±0.032	0.610±0.043
	AJCC	0.615±0.033	0.632±0.058		0.598±0.029	0.587±0.047
	BCLC	0.645±0.035	0.678±0.060		0.617±0.030	0.597±0.050
	CLIP	0.634±0.037	0.669±0.061		0.606±0.032	0.592±0.051

AIC, Akaike information criterion; ALBI, Albumin-bilirubin; GGT, Gamma-glutamyl transpeptidase; AJCC, American Joint Committee on Cancer, BCLC, Barcelona Clinic Liver Cancer; CLIP, Cancer of the Liver Italian Program.

## Abbreviations

ALBI: albumin-bilirubin
GGT: serum γ-glutamyl transpeptidase
OS: overall survival
DFS: disease-free survival
HCC: hepatocellular carcinoma
AJCC: American Joint Commission on Cancer
BCLC: Barcelona Clinic Liver Cancer
CLIP: Cancer of the Liver Italian Program
AICs: Akaike information criterion
C-P: Child-Pugh
C-index: Concordance index
